# Special Issue: Frontiers in RNA Structure

**DOI:** 10.3390/molecules25204843

**Published:** 2020-10-21

**Authors:** Quentin Vicens

**Affiliations:** Department of Biochemistry and Molecular Genetics, University of Colorado Denver School of Medicine, Aurora, CO 80045, USA; QUENTIN.VICENS@UCDENVER.EDU; Tel.: +1-303-724-3256

The frontiers of our knowledge about RNA structure are rapidly moving. Twenty-five years ago, there were fewer RNA structures in total than there are generally today within a single family of RNAs (such as tRNAs, riboswitches, or ribozymes). The longest RNA ever solved had 160 nucleotides [1]. Today, structures of ribonucleoproteins (RNPs) with RNAs that have lengths of several hundreds or thousands of nucleotides are released on a weekly basis.

Constantly pushing technical boundaries is what enabled us to generate, analyze and make sense of this sheer amount of structural information. In turn, the triumph of methods such as single-molecule studies or cryo-electron microscopy (cryo-EM) has offered glimpses into the function of large RNP assemblies. We have reached a point where we have three-dimensional visuals of the various stages along core cellular processes like transcription and translation [2,3].

However, we are not done. The field of RNA structural biology has never been so vibrant. The current COVID-19 pandemic naturally brought an impetus to advance the field even further, as vaccines may be available one day that will be RNA-based, or drugs might be developed that target an RNA genome. In some way, tackling RNA viruses epitomizes where the field is at: the study of long RNAs that adopt transient structures, each having distinct regulatory roles through binding small molecules, proteins, DNA, or other RNAs.

This Special Issue of *Molecules* lies precisely at the intersection between the biology of RNAs acting as sensors or regulators, and the methods we are developing to study them.

Zappulla at Lehigh University and Bou-Nader and Zhang at NIH propose timely reviews on long RNAs, how they may fold as “flexible scaffolds” to spatially but loosely constrain binding partners [4], and how they may interact with one another, leading to intricate quaternary structures [5]. In the cell, some of these RNA molecules may drive the formation of P-bodies and stress granules by interacting with one another and with proteins, as reviewed by the Trcek lab at Johns Hopkins [6].

Long RNAs nonetheless comprise independently folded modules. Using X-ray crystallography, the Batey lab at the University of Colorado delivers a thought-provoking story that will help shift the paradigm of ligand binding to riboswitches located upstream of messenger RNA in bacteria [7]. The Kondo lab from Sophia University in Japan proposes a structure-based rationale for why certain point mutations are not tolerated in the ribosome decoding site [8].

Fleeting RNPs may be characterized by single-molecule fluorescence approaches, as reviewed by the Hengesbach lab at Goethe University, Frankfurt [9]. They illustrate the power of such methods to sort out the mechanism of complex and dynamic RNPs like the spliceosome and the assemblies that maintain the integrity of telomeres. Cryo-EM enables us to visualize dynamic architectures, as portrayed by the Dao Duc laboratory at the University of British Columbia, who review ribosome heterogeneity [10]. In the same vein, the teams led by Marcoux, Gleizes and Plisson-Chastang at the University of Toulouse in France determine the role of a few of the many ribosome biogenesis factors, demonstrating the power of cryo-EM to analyze conformational heterogeneity [11].

I am very excited about the collection of articles in this Special Issue for two reasons. First, constantly updating our perception of RNA folding and structure would not be possible without concomitant advances in biophysical approaches, as this collection of reviews and original work exemplifies. Second, the various labs that have deemed it worth their precious time to submit a manuscript are esteemed colleagues, ranging from long-time collaborators to serendipitous encounters. The majority represent young principal investigators.
